# A case of canalicular adenoma with anemia

**DOI:** 10.1093/jscr/rjab606

**Published:** 2022-02-15

**Authors:** Yuko Komatsu, Tadashi Kawai, Toshimi Chiba, Yasunori Takeda, Hiroyuki Yamada

**Affiliations:** Division of Oral and Maxillofacial Surgery, Department of Oral and Maxillofacial Reconstructive Surgery, School of Dentistry, Iwate Medical University, Morioka, Iwate, Japan; Division of Oral and Maxillofacial Surgery, Department of Oral and Maxillofacial Reconstructive Surgery, School of Dentistry, Iwate Medical University, Morioka, Iwate, Japan; Division of Internal Medicine, Department of Oral Medicine, School of Dentistry, Iwate Medical University, Morioka, Iwate, Japan; Division of Clinical Pathology, Department of Oral and Maxillofacial Reconstructive Surgery, School of Dentistry, Iwate Medical University, Shiwa-gun, Iwate, Japan; Division of Oral and Maxillofacial Surgery, Department of Oral and Maxillofacial Reconstructive Surgery, School of Dentistry, Iwate Medical University, Morioka, Iwate, Japan

## Abstract

An 81-year-old woman presented with bleeding from a soft elastic bulge on the right buccal mucosa. Her medical history included hypertension, chronic renal failure, hysterectomy and a goiter operation. Certain investigations, such as blood tests, which confirmed the minimum hemoglobin level of 5.3 g/dl, computed tomography and magnetic resonance imaging (MRI), were conducted. MRI revealed features confirming the presence of a tumor involving the right buccal mucosa with high signal in T2-enhanced image. Considering the provisional diagnosis and image findings, the tumor mass was excised and histopathological examination of the biopsy specimen confirmed the diagnosis of a canalicular adenoma. Under the diagnosis of various clinical departments, the anemic state of the patient might be chiefly attributed to the bleeding from the tumor. Herein, we report a rare case of a canalicular adenoma in the right buccal mucosa with anemia due to bleeding from the tumor.

## INTRODUCTION

In 1953, canalicular adenoma (CA), a rare benign salivary gland tumor [1, 2]. Salivary gland tumors constitute 2–3% of all neoplasms in the head and neck. Further, CA is detected in 1% of all the salivary gland neoplasms and 4% of minor salivary gland tumors [3, 4]. The most common site of its occurrence is the upper lip, accounting for 80% of this tumor, followed by the buccal mucosa [5, 6]. The incidence of CA is greatest among women aged 70 years. Microscopically, the tumors are bosselated or lobulated with a well-circumscribed periphery. Majority of the cases display cyst formation, frequently accompanied by intraluminal hemorrhage [2]. Although the tumor has abundant luminal structure, there are no reports of adverse effects due to bleeding from the tumor. Herein, we report a case of CA in the right buccal mucosa of an 81-year-old woman with severe anemia due to bleeding of the tumor.

## CASE REPORT

### Clinical features

An 81-year-old woman presented to our hospital with bleeding from the right buccal mucosa. The patient had a history of chronic kidney failure. At presentation the hemoglobin (Hb) level was 6.7 g/dl.

**Figure 1:**
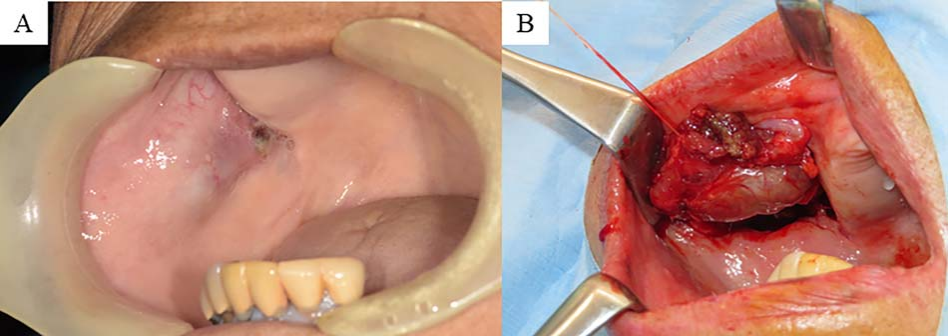
(**A**) Intraoral findings: bulge sized 30 mm, soft and elastic in consistency, involving the right buccal mucosa. (**B**) Intraoperative findings: the tumor is covered with a white capsule, and the border with the surrounding organization is clear.

**Figure 2:**
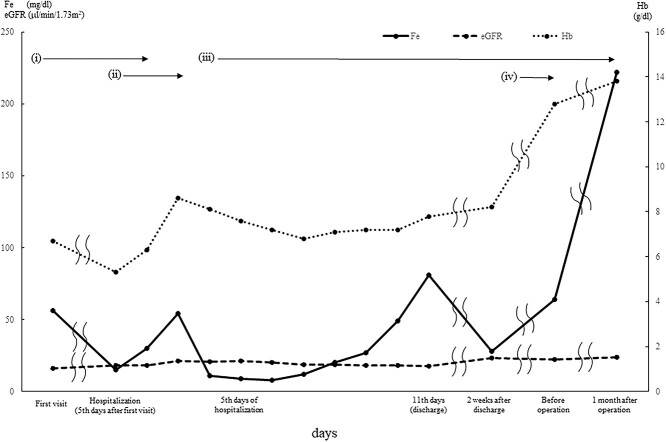
Blood test findings: the Hb concentration level improved on blood transfusion; however, a decline in Hb or iron levels is seen after the third day of hospitalization. Administration of iron and erythropoietin improved Hb and iron levels. Due to renal anemia, estimated glomerular filtration rate level remained low. (i) Continuous bleeding. (ii) Blood transfusion of four units of red cell concentrate mannitol adenine phosphate for 2 days. (iii) Administration of iron. (iv) Administration of erythropoietin.

The next day, the patient was referred to our outpatient department. The extraoral examination was normal, intraoral examination revealed a soft elastic bulge in the right buccal mucosa measuring 30 mm in diameter (Fig. 1A). The bleeding from the right buccal mucosa was detected daily, and the anemia progressed. The patient’s iron level was also low after bleeding, and oral administration of iron was initiated. On the fifth day after the first medical examination, her Hb level was confirmed to be 5.3 g/dl. The changes in the blood test results are shown in Fig. 2. The patient was hospitalized on the same day, and we performed blood transfusion of two units of red cell concentrate mannitol adenine phosphate on the first and second day of hospitalization. And on the second day, we performed biopsy form the bleeding point and sutured closely to make the bleeding to stop. However, The Hb level reduced again within a few days. The histopathological diagnosis was confirmed as CA, and there was no evidence of malignancy.

We considered four possible reasons for the persistent anemia after blood transfusion. First, re-bleeding from the tumor. Hence, we clearly located the bleeding point, and eventually the bleeding stopped. Second, the presence of renal anemia; hence, the patient was referred to the department of urology for further examination, and the findings also indicated renal anemia. Third, gastrointestinal bleeding as we could not detect any bleeding point in the oral cavity. However, the presence of blood in stool and hematemesis was not detected, and the possibility of gastrointestinal bleeding was low. Lastly, we considered the presence of a blood disorder, which was further investigated by the department of hematology and oncology. To exclude other diseases, certain investigations were conducted; however, all yielded negative results. The patient was discharged on Day 11 of hospitalization. Her Hb level at the time of discharge was 7.8 g/dl. The iron level remained low.

### Image findings

**Figure 3:**
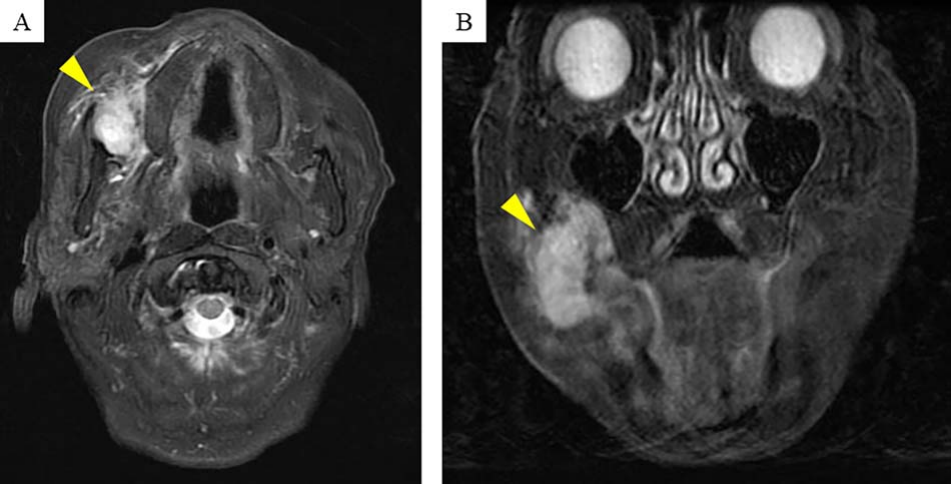
Magnetic resonance imaging (MRI) findings: contrast-enhanced T2-weighted MRI shows a high signal mass (arrow head) of size 31 × 25 × 30 mm in the right buccal mucosa.

Computerized tomography showed a mass, measuring 31 × 25 × 30 mm, located in the right buccal mucosa. On magnetic resonance imaging, contrast-enhanced T2-weighted images showed a high signal (Fig. 3A and B).

### Treatment progress

Owing to the vascularity of the tumor, there were concerns regarding the risk of re-bleeding from the tumor and aggravation of symptoms related to anemia. Therefore, we planned tumor resection, which was conducted within 5 months after the discharge. Before surgery, we administered an erythropoietin-based treatment for renal anemia. The tumor was covered with a capsule and clearly bordered by the surrounding tissue (Fig. 1B). The Hb level was maintained at ~12 g/dl as confirmed by a postoperative blood test. The aggravation of anemia was not detected for 3 months after operation.

### Histopathological findings

**Figure 4:**
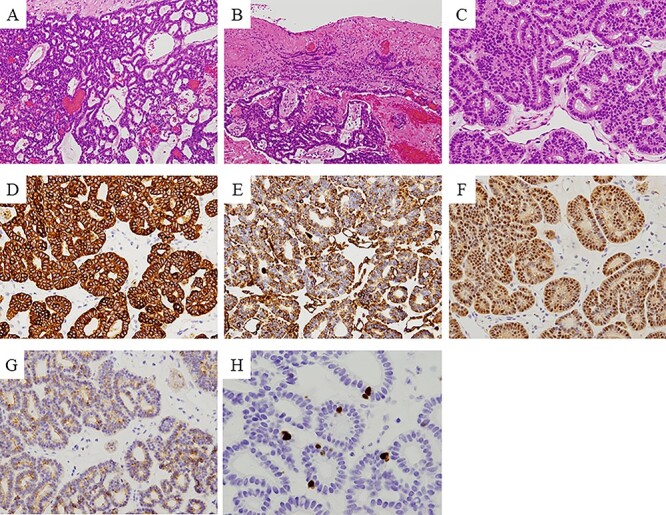
Immunohistochemical findings: the lesion is a well-circumscribed and encapsulated tumor mass (**A**), but the overlying mucosa is discontinuous in some areas caused by chronic traumatic irritation with recurrent hemorrhage (**B**). The tumor is composed of proliferating epithelial cells basically arranged in branching and interconnecting canalicular strands or cords lined by a single-layered columnar cells, and these canalicular structures dilate in various degrees resulting in formation of cystic structures (A, **C**). Immunohistochemical examination revealed that the tumor cells were positive for 34βE12, vimentin, S100 and c-KIT (**D**–**G**), and negative for SMA and p63. Ki-67 positive cells were less than 5% per high-power view field (**H**).

The lesion was a well-circumscribed and encapsulated tumor mass (Fig. 4A), but the overlying mucosa appeared discontinuous in some areas due to chronic traumatic irritation with repeated episodes of hemorrhage (Fig. 4B). The tumor mass comprised proliferating epithelial cells arranged in branching and interconnecting canalicular strands or cords lined by single-layered columnar cells, and these canalicular structures dilated to various degrees, resulting in the formation of cystic structures (Fig. 4A and C).

Immunohistochemical examination revealed that the tumor cells were positive for 34βE12, vimentin, S100 and c-KIT (Fig. 4D–G), and negative for SMA and p63. Ki-67 positive cells were < 5% per high-power field (Fig. 4H).

Based on these histopathological and immunohistochemical findings, the lesion was diagnosed as CA.

## DISCUSSION

CA is a rare benign salivary gland tumor. Previous reports have no case of anemia due to bleeding from a CA is an extremely rare finding and has not been reported before.

We considered the possibility of iron deficiency anemia [7]. Since the associated anemia was categorized as normocytic anemia, the main cause of anemia was more likely to be related to bleeding [8]. Thus, red blood cell transfusion seemed to temporarily improve the Hb. The associated sudden drop in iron level also improved with blood transfusion on the fourth day of hospitalization. The iron level had gradually decreased, and the Hb followed a similar trend. Hence, iron administration was initiated, and the recovery of iron level was observed from the eighth day of hospitalization, and Hb also improved accordingly. Replacement therapy with erythropoietin was started because of the low erythropoietin level associated with chronic kidney failure [9], and the Hb concentration improved significantly afterward. Further, symptoms related to anemia improved subsequent to the decrease in bleeding from the tumor. The consumptive iron deficiency due to bleeding was related to the anemia and progressed slowly after blood transfusion. This improved through the intake of iron supplements and erythropoietin therapy.

In patients having some factors that induce progression of severe anemia, including bleeding from a tumor, similar to this case. Therefore, if the tumor is benign, but at risk of bleeding, aggressive resection should be considered.
